# Supplementary material for: Bacterial Communities in the Rhizosphere and Phyllosphere of Halophytes and Drought-Tolerant Plants in Mediterranean Ecosystems
Source: Microorganisms. 2020 Oct 31;8(11):1708. doi: 10.3390/microorganisms8111708 (PMC7692439; doi:10.3390/microorganisms8111708)
Supplement: Supplementary file 1 [file microorganisms-08-01708-s001.zip › Supplementary material/Table S1.docx]

**Supplementary Table S1.** Number of OTUs, the richness estimator (S_Chao1_), the ratio observed / expected OTUs and the heterogeneity of the alpha-diversity indexes (the Simpson dominance and Equitability indexes) and the total number of reads in each sample. The total and average number of OTUs, number of reads and average values of the alpha diversity estimators are also shown. N/A: Not Applicable.

| **Code** | **Nb of OTUs** | **S_Chao1_** | **Observed / Expected OTUs** | **Simpson (1-*D*)** | **Equitability (H/H_max_)** | **Nb of reads** |
| --- | --- | --- | --- | --- | --- | --- |
| VlRz1 | 486 | 559 | 0.87 | 0.98 | 0.79 | 4134 |
| VlRz2 | 967 | 1444 | 0.67 | 0.97 | 0.78 | 5554 |
| VlRz3 | 593 | 871.2 | 0.68 | 0.96 | 0.73 | 4509 |
| VlRz4 | 747 | 995.6 | 0.75 | 0.97 | 0.75 | 5773 |
| VlRz5 | 336 | 352.4 | 0.95 | 0.99 | 0.86 | 2945 |
| VlRz6 | 908 | 1143 | 0.79 | 0.99 | 0.86 | 6305 |
| VlRz7 | 645 | 759.1 | 0.85 | 0.99 | 0.85 | 4212 |
| VlRz8 | 361 | 509.8 | 0.71 | 0.98 | 0.82 | 2847 |
| **Total** | **2316** |  |  |  |  |  |
| **Average ± SD** | **630.38 ±**  **219.56** | **829.26 ± 337.86** | **0.78 ± 0.09** | **0.98 ± 0.01** | **0.81 ± 0.05** | **4534.88 ± 1190.96** |
| VlPh1 | 503 | 578 | 0.87 | 0.95 | 0.70 | 6734 |
| VlPh2 | 207 | 210.1 | 0.99 | 0.98 | 0.87 | 2604 |
| VlPh3 | 185 | 194.8 | 0.95 | 0.98 | 0.86 | 1292 |
| VlPh4 | 208 | 262.6 | 0.79 | 0.77 | 0.44 | 6659 |
| VlPh5 | 97 | 150 | 0.65 | 0.07 | 0.07 | 6951 |
| VlPh6 | 123 | 150 | 0.82 | 0.50 | 0.39 | 2226 |
| VlPh7 | 108 | 111.7 | 0.97 | 0.95 | 0.86 | 728 |
| VlPh8 | 354 | 385 | 0.92 | 0.98 | 0.86 | 2443 |
| **Total** | **1272** |  |  |  |  |  |
| **Average ± SD** | **223.13 ± 130.67** | **255.28 ± 145.56** | **0.87 ± 0.11** | **0.77 ± 0.31** | **0.63 ± 0.28** | **3704.63 ± 2452.42** |
| EmpRz1 | 570 | 666.7 | 0.85 | 0.98 | 0.77 | 6607 |
| EmpRz2 | 882 | 1071 | 0.82 | 0.99 | 0.85 | 6613 |
| EmpRz3 | 877 | 1071 | 0.82 | 0.99 | 0.84 | 6571 |
| EmpRz4 | 1015 | 1838 | 0.55 | 0.98 | 0.78 | 6968 |
| EmpRz5 | 1161 | 1819 | 0.64 | 0.99 | 0.81 | 6922 |
| EmpRz6 | 945 | 1238 | 0.76 | 0.99 | 0.82 | 6893 |
| EmpRz7 | 1046 | 1703 | 0.61 | 0.98 | 0.78 | 6804 |
| EmpRz8 | 1455 | 2374 | 0.61 | 0.99 | 0.85 | 6978 |
| **Total** | **3271** |  |  |  |  |  |
| **Average ± SD** | **993.88 ± 237.69** | **1472.59 ± 517.95** | **0.71 ± 0.11** | **0.99 ± 0.01** | **0.81 ± 0.03** | **6794.5 ± 161.2** |
| EmpPh1 | 360 | 372.1 | 0.97 | 0.98 | 0.87 | 3676 |
| EmpPh2 | 578 | 626 | 0.92 | 0.99 | 0.89 | 5062 |
| EmpPh3 | 406 | 413.3 | 0.98 | 0.99 | 0.89 | 3650 |
| EmpPh4 | 1107 | 1504 | 0.74 | 0.99 | 0.86 | 6221 |
| EmpPh5 | 967 | 1209 | 0.80 | 0.99 | 0.84 | 6241 |
| EmpPh6 | 825 | 1052 | 0.78 | 0.98 | 0.82 | 5686 |
| EmpPh7 | 1436 | 2467 | 0.58 | 0.99 | 0.85 | 6819 |
| EmpPh8 | 949 | 1192 | 0.80 | 0.99 | 0.87 | 5148 |
| **Total** | **3112** |  |  |  |  |  |
| **Average ± SD** | **828.5 ± 342.98** | **1104.43 ± 640.36** | **0.82 ± 0.12** | **0.99 ± 0** | **0.86 ± 0.02** | **5312.88 ± 1097.06** |
| MenRz1 | 577 | 1025 | 0.56 | 0.93 | 0.64 | 7110 |
| MenRz2 | 1027 | 1374 | 0.75 | 0.99 | 0.86 | 6975 |
| MenRz3 | 1252 | 1702 | 0.74 | 0.99 | 0.88 | 6984 |
| MenRz4 | 1469 | 2463 | 0.60 | 0.99 | 0.88 | 7069 |
| MenRz5 | 805 | 873.6 | 0.92 | 0.99 | 0.87 | 7078 |
| **Total** | **2745** |  |  |  |  |  |
| **Average ± SD** | **1026 ± 315.52** | **1487.52 ± 565.91** | **0.71 ± 0.13** | **0.98 ± 0.02** | **0.82 ± 0.09** | **7043.20 ± 53.84** |

| **Code** | **Nb of OTUs** | **S_Chao1_** | **Observed / Expected OTUs** | **Simpson (1-*D*)** | **Equitability (H/H_max_)** | **Nb of reads** |
| --- | --- | --- | --- | --- | --- | --- |
| MenPh1 | 248 | 252.4 | 0.98 | 0.97 | 0.82 | 3452 |
| MenPh2 | 219 | 223 | 0.98 | 0.85 | 0.68 | 4712 |
| MenPh3 | 164 | 172.1 | 0.95 | 0.93 | 0.74 | 4577 |
| MenPh4 | 289 | 368.6 | 0.78 | 0.90 | 0.57 | 6994 |
| **Total** | **613** |  |  |  |  |  |
| **Average ± SD** | **230 ± 45.5** | **254.03 ± 72.12** | **0.93 ± 0.07** | **0.92 ± 0.04** | **0.70 ± 0.09** | **4933.75 ± 1286.14** |
| CisRz1 | 1186 | 1472 | 0.81 | 0.99 | 0.87 | 7076 |
| CisRz2 | 586 | 594 | 0.99 | 0.99 | 0.89 | 5499 |
| CisRz3 | 1008 | 1287 | 0.78 | 0.99 | 0.85 | 7095 |
| CisRz4 | 1171 | 1362 | 0.86 | 0.99 | 0.89 | 7063 |
| **Total** | **2436** |  |  |  |  |  |
| **Average ± SD** | **987.75 ± 242.23** | **1178.75 ± 343.96** | **0.86 ± 0.08** | **0.99 ± 0** | **0.88 ± 0.02** | **6683.25 ± 683.82** |
| CisPh1 | 388 | 409 | 0.95 | 0.97 | 0.75 | 7124 |
| ThRz1 | 1577 | 2735 | 0.58 | 0.99 | 0.85 | 7048 |
| ThRz2 | 1192 | 2064 | 0.58 | 0.99 | 0.80 | 6948 |
| ThRz3 | 1249 | 1952 | 0.64 | 0.99 | 0.85 | 6941 |
| ThRz4 | 821 | 1098 | 0.75 | 0.99 | 0.80 | 7066 |
| **Total** | **3089** |  |  |  |  |  |
| **Average ± SD** | **1209.75 ± 268.26** | **1962.25 ± 581.92** | **0.64 ± 0.07** | **0.99 ± 0** | **0.83 ± 0.03** | **7000.75 ± 56.66** |
| ThPh1 | 312 | 324.5 | 0.96 | 0.98 | 0.83 | 6889 |
| ThPh2 | 291 | 299.4 | 0.97 | 0.97 | 0.80 | 6029 |
| ThPh3 | 677 | 1048 | 0.65 | 0.97 | 0.73 | 7099 |
| ThPh4 | 134 | 137.5 | 0.97 | 0.97 | 0.85 | 3256 |
| **Total** | **993** |  |  |  |  |  |
| **Average ± SD** | **353.50 ± 199.04** | **452.35 ± 351.31** | **0.89 ± 0.11** | **0.97 ± 0** | **0.8 ± 0.05** | **5818.25 ± 1532.67** |
| SarRz1 | 1238 | 2004 | 0.62 | 0.99 | 0.84 | 7036 |
| SarRz2 | 1386 | 2287 | 0.61 | 1.00 | 0.87 | 7054 |
| SarRz3 | 1245 | 1979 | 0.63 | 0.99 | 0.85 | 7068 |
| SarRz4 | 824 | 1105 | 0.75 | 0.99 | 0.84 | 7079 |
| SarRz5 | 780 | 1239 | 0.63 | 0.99 | 0.80 | 7104 |
| **Total** | **2806** |  |  |  |  |  |
| **Average ± SD** | **1094.6 ± 245.07** | **1722.8 ± 464.49** | **0.65 ± 0.05** | **0.99 ± 0** | **0.84 ± 0.02** | **7068.2 ± 22.96** |
| SarPh1 | 377 | 480.4 | 0.78 | 0.96 | 0.70 | 6835 |
| SarPh2 | 525 | 606.6 | 0.87 | 0.99 | 0.80 | 6809 |
| SarPh4 | 310 | 318.1 | 0.97 | 0.98 | 0.84 | 4917 |
| SarPh5 | 309 | 323.8 | 0.95 | 0.98 | 0.81 | 6986 |
| **Total** | **978** |  |  |  |  |  |
| **Average ± SD** | **380.25 ± 88** | **432.23 ± 119.9** | **0.89 ± 0.08** | **0.98 ± 0.01** | **0.79 ± 0.05** | **6386.75 ± 851.25** |
| CrRz1 | 1078 | 1294 | 0.83 | 0.99 | 0.86 | 7055 |
| CrRz2 | 1071 | 1273 | 0.84 | 1.00 | 0.89 | 6975 |
| CrRz3 | 1356 | 1796 | 0.76 | 1.00 | 0.89 | 7043 |
| CrRz4 | 953 | 1074 | 0.89 | 0.99 | 0.88 | 6998 |
| CrRz5 | 1255 | 1617 | 0.78 | 1.00 | 0.88 | 7032 |
| **Total** | **3260** |  |  |  |  |  |
| **Average ± SD** | **1142.6 ± 143.82** | **1410.80 ± 259.6** | **0.82 ± 0.05** | **0.99 ± 0** | **0.88 ± 0.01** | **7020.6 ± 29.68** |

| **Code** | **Nb of OTUs** | **S_Chao1_** | **Observed / Expected OTUs** | **Simpson (1-*D*)** | **Equitability (H/H_max_)** | **Nb of reads** |
| --- | --- | --- | --- | --- | --- | --- |
| CrPh2 | 60 | 69.17 | 0.87 | 0.60 | 0.41 | 4877 |
| CrPh3 | 71 | 75.5 | 0.94 | 0.82 | 0.56 | 5488 |
| **Total** | **118** |  |  |  |  |  |
| **Average** | **65.5** | **72.34** | **0.9** | **0.71** | **0.48** | **5182.50** |
| AtrRz1 | 1277 | 1788 | 0.71 | 1.00 | 0.87 | 7022 |
| AtrRz2 | 996 | 1400 | 0.71 | 0.99 | 0.85 | 7060 |
| AtrRz3 | 1163 | 1610 | 0.72 | 0.99 | 0.85 | 7062 |
| AtrRz4 | 1203 | 1856 | 0.65 | 0.99 | 0.84 | 7092 |
| AtrRz5 | 1161 | 1708 | 0.68 | 0.99 | 0.85 | 7025 |
| **Total** | **3064** |  |  |  |  |  |
| **Average ± SD** | **1160 ± 92.13** | **1672.4 ± 159.02** | **0.7 ± 0.03** | **0.99 ± 0** | **0.85 ± 0.01** | **7052.2 ± 26.05** |
| AtrPh1 | 462 | 488 | 0.95 | 0.98 | 0.85 | 6946 |
| AtrPh5 | 132 | 136.1 | 0.97 | 0.93 | 0.74 | 2780 |
| **Total** | **552** |  |  |  |  |  |
| **Average** | **297** | **312.05** | **0.96** | **0.95** | **0.80** | **4863** |
