# Supplementary figures and images for: Bacterial Communities in the Rhizosphere and Phyllosphere of Halophytes and Drought-Tolerant Plants in Mediterranean Ecosystems
Source: Microorganisms. 2020 Oct 31;8(11):1708. doi: 10.3390/microorganisms8111708 (PMC7692439; doi:10.3390/microorganisms8111708)

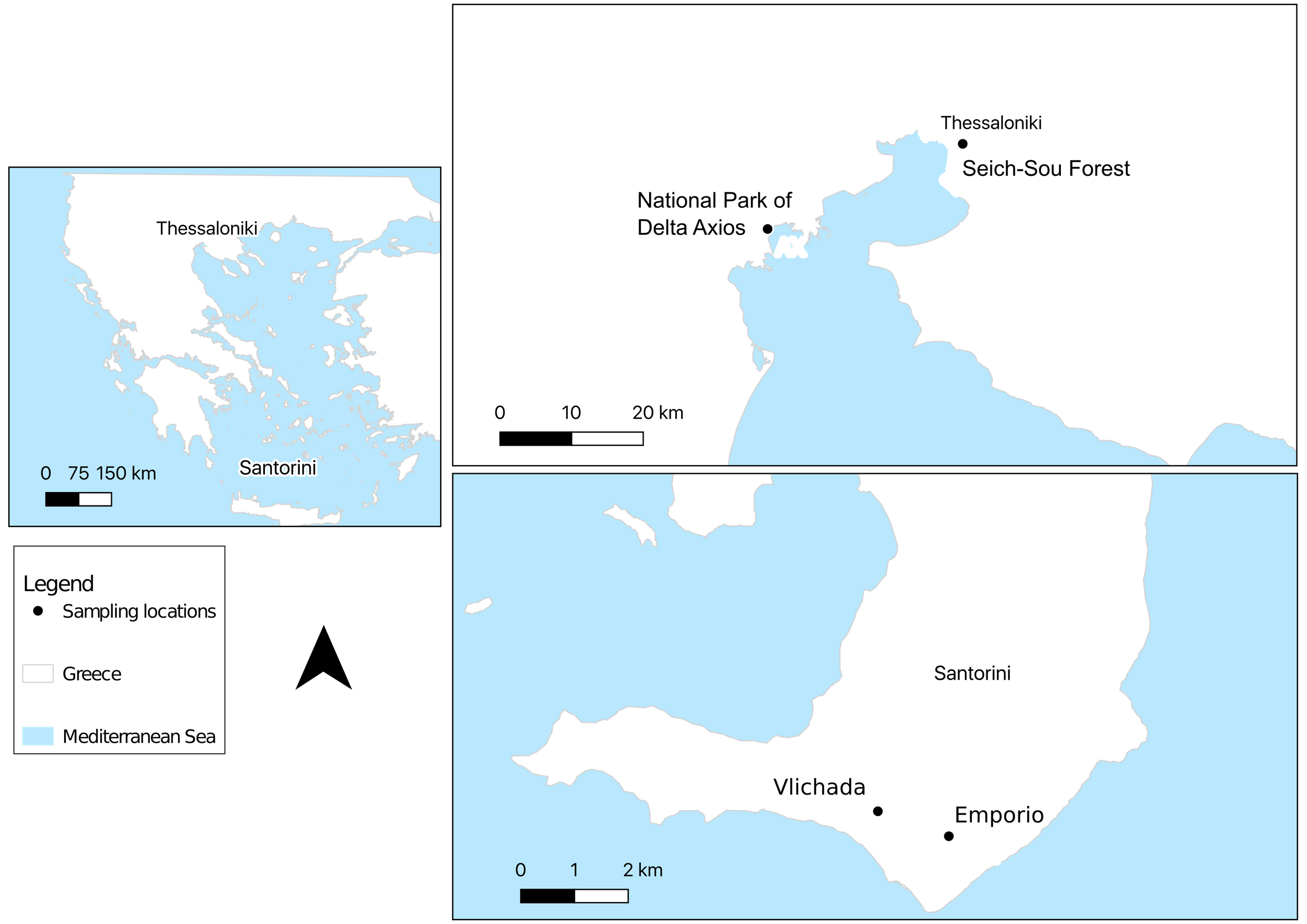

Supplement: Supplementary file 1 [file microorganisms-08-01708-s001.zip › Supplementary material/Figure S1.jpg]

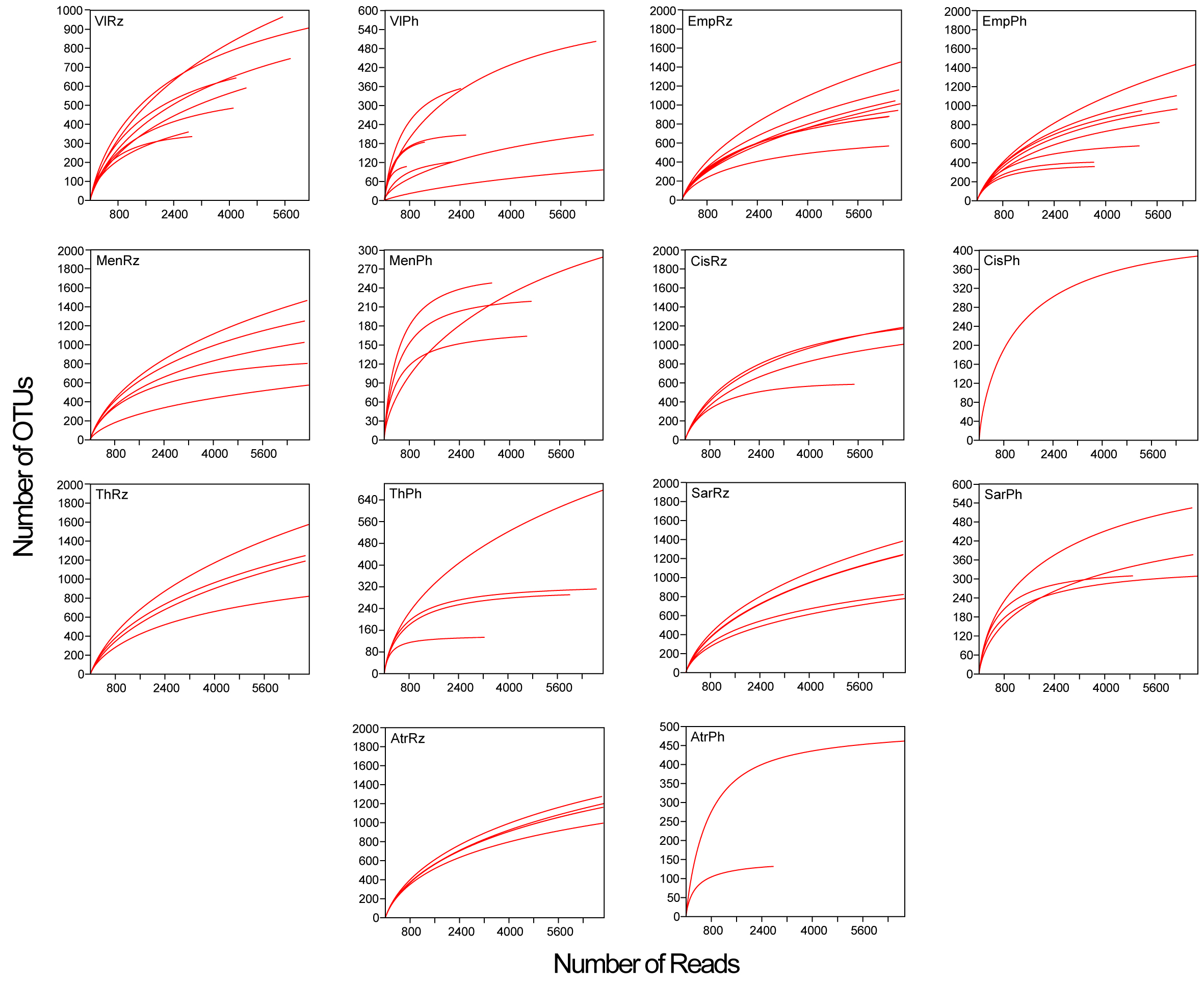

Supplement: Supplementary file 1 [file microorganisms-08-01708-s001.zip › Supplementary material/Figure S2.jpg]
